# Enacting Proprioceptive Predictions in the Rubber Hand Illusion

**DOI:** 10.3389/fnhum.2022.839890

**Published:** 2022-02-16

**Authors:** Jakub Limanowski

**Affiliations:** ^1^Faculty of Psychology, Technische Universität Dresden, Dresden, Germany; ^2^Centre for Tactile Internet With Human-in-the-Loop, Technische Universität Dresden, Dresden, Germany

**Keywords:** action, active inference, predictive coding, proprioceptive drift, rubber hand illusion

In the “rubber hand illusion,” the participant sees a displaced fake hand being touched congruently with her unseen real hand. This seems to invoke inference of an “illusory” common cause for visual, tactile, and proprioceptive sensations; as evident from a perceived embodiment of the fake hand and the perception of one's unseen hand location closer toward the position of the fake hand—the so-named “proprioceptive drift.” Curiously, participants may sometimes *move* their hand in the direction of the fake hand (Asai, 2015). While this could easily be explained as participants actively trying to align the real and fake hands to experience a stronger illusion, they are *not aware* of these movements (cf. Abdulkarim and Ehrsson, 2018). So there may be better explanation for this observation than that participants were “cheating.” In their recent article, Lanillos et al. (2021) show that the unintentional execution of arm movement forces during a virtual reality based version of the rubber hand illusion—which the authors call “active drift”—can be reproduced by a computational model based on the *active inference* framework.

Active inference can be described as an extension of predictive coding schemes; i.e., based on the assumption that agents entail and optimize a generative model of the hidden causes of their sensations (Friston et al., 2011). Put simply, the model's “beliefs” (probabilistic representations) capture statistical regularities in the environment and are, in turn, optimized by accommodating prediction errors. The optimisation of model beliefs by prediction errors, to match the sensory data, corresponds to perceptual inference. *Active* inference extends this idea to include movement and action. This opens up another way for the agent to deal with prediction errors; namely, acting on the environment to directly reduce them (Palmer et al., 2016). One of the unique, and controversial, assumptions of such an active inference formulation is that movement is the “enaction” of *proprioceptive predictions* by the motor system. Thus, motor commands are described as proprioceptive predictions about the state of the body, generated by optimized beliefs in the motor cortex (Adams et al., 2013). The peripheral motor system is then thought to minimize the error between predicted and actual proprioceptive states; e.g., through spinal reflex arcs (Friston et al., 2011).

Leveraging this approach, Lanillos et al. (2021) were able to simulate real-hand forces exerted in the direction of the fake hand in terms of attempted movements (i.e., measured by applied force, while actual movement was precluded) driven by the minimization of proprioceptive prediction errors. In other words, these forces could be explained as “enactions” of proprioceptive predictions of a generative model that jointly predicts visual and proprioceptive cues and, thus, the most likely body position. These results are an impressive validation of some of active inference's key assumptions about perception-action coupling.

Lanillos et al.'s approach can and should be taken further to address some unanswered questions about bodily illusions and visuo-proprioceptive conflicts. For instance, active inference offers a new perspective on the (not yet fully clear) relationship between the “proprioceptive drift” and the “active drift” in the rubber hand illusion.

Previous computational models of the rubber hand illusion in terms of perceptual inference (Samad et al., 2015; Hinz et al., 2018) were successfully used to simulate the proprioceptive drift. Within predictive coding schemes, the proprioceptive drift could be related to accommodating proprioceptive prediction errors—arising from the mismatch between the estimated hand location (biased by the illusion) and actual proprioceptive signals—by updating the model's proprioceptive beliefs (cf. Hinz et al., 2018). While this corresponds to prediction error accommodation through adjusting *perception*, the forces simulated by Lanillos et al. suggest that proprioceptive prediction errors resulting from the rubber hand illusion can also directly be minimized through *action*; i.e., by changing the proprioceptive data so that they correspond to the model's beliefs.

Proprioceptive drift was neither explicitly modeled nor measured in the empirical sample by Lanillos et al. However, the forces were triggered by an analogous mechanism—proprioceptive prediction error minimization—that the authors had previously used to simulate the proprioceptive drift (Hinz et al., 2018). Correspondingly, the authors speculate that “both effects may be driven by the same underlying process” (Lanillos et al., 2021, p. 8). This speculation is supported by the empirical results of Asai (2015), who found that proprioceptive drifts and forces applied during the rubber hand illusion, indeed, correlated *positively* (albeit weakly) in human subjects.

Following the active inference framework's conceptualization of movements as being driven by proprioceptive predictions, both phenomena can be formulated as arising from the same mechanism; namely, updating proprioceptive representations (“beliefs”) of one's hand location. The *proprioceptive* drift could thus be seen as a perceptual proxy of the recalibration of proprioceptive beliefs, and the *active* drift as an attempted “enaction” of the new hand location predicted by this recalibration.

Furthermore, active inference proposes a different mechanism that could also influence the relationship between the proprioceptive and active drifts; namely, *somatosensory attenuation*. Sensory attenuation means, loosely speaking, an attenuation of the impact that some sensory input has on higher-level neuronal processes or perception, potentially implemented in terms of a reduction of the *precision* (inverse variance) of the corresponding sensory signals (Brown et al., 2013; Palmer et al., 2016). Notably, along active inference, somatosensory attenuation also is a prerequisite for movement initiation. As noted, motor “commands” are formulated as proprioceptive predictions about the state of the body. Initially, these predictions conflict with sensory evidence received from the body—which is consequently treated as a prediction error. Therefore, the weight of sensory evidence relative to model predictions is temporarily attenuated. This allows model predictions to dominate; i.e., to initiate and drive movement toward the predicted body state (Brown et al., 2013; Palmer et al., 2016).

Somatosensory attenuation may be crucial for understanding the rubber hand illusion, since like many other multisensory phenomena, the illusion fundamentally depends on the relative precision afforded to the respective sensory cues. I.e., since vision has a higher sensory precision than proprioception, the seen fake hand position typically “captures” the unseen real hand's one. This visual dominance over proprioception—and thus, also the proprioceptive drift—can be enhanced by somatosensory attenuation; i.e., attenuating proprioceptive precision [see Limanowski (2021), for a review of empirical evidence for this argument].

One could, therefore, speculate that in the rubber hand illusion, somatosensory attenuation is initially deployed to assist multisensory integration and sensory recalibration under visuo-proprioceptive conflict; i.e., by suppressing proprioceptive evidence relative to vision. This would implicitly lower the weight of proprioceptive evidence vs proprioceptive predictions; which in turn could result in a lower threshold for (involuntary) movement initiation toward the recalibrated own hand location—i.e., an active reduction of prediction error between the predicted and the sensed hand position.

This is speculation at this point, and there are alternative interpretations. In principle, it is also possible to conceptualize the active drift as “an *alternative* to ‘passive' perceptual recalibrations such as the proprioceptive drift” (Lanillos et al., 2021, p. 2, emphasis added). Per analogy, the decision on whether to move or not may depend on whether I ignore (attenuate) sensory evidence suggesting I am stationary, and let model beliefs initiate a movement (changing the world through action to fit model beliefs); *or* I let the sensory evidence update my model beliefs, remaining stationary (Palmer et al., 2016). Thus, in the rubber hand illusion, one could also expect that participants reporting a stronger proprioceptive drift would exhibit a *smaller* active drift—not “needing to move” as much, because the proprioceptive prediction error was already accommodated by perceptual inference (adjusted model beliefs now already represent the proprioceptive hand position closer to the visual one).

Thus, more work is needed to clarify how exactly sensory attenuation, proprioceptive recalibration, and the involuntary production of forces or movements relate. Clarifying this relationship is important because it could, among other things, contribute to a deeper understanding of phenomena such as the tendency to automatically imitate observed actions (e.g., Brass et al., 2001), which have gained a new importance in light of recent developments in virtual reality (Kokkinara et al., 2016; Burin et al., 2020; Gonzalez-Franco et al., 2020). While these are mostly empirical questions, computational models based on active inference can be developed further, to disambiguate between the above possibilities. For instance, using a relatively simple active inference model, we could relate the interference of observed virtual hand movements with actual movement execution to attentional effects (Limanowski and Friston, 2020). The study by Lanillos et al. is an important step into the right direction that shows how conceptual and empirical questions can jointly be addressed with an active inference formulation.

## Author Contributions

The author confirms being the sole contributor of this work and has approved it for publication.

## Funding

This work was funded by a Freigeist Fellowship of the VolkswagenStiftung (AZ 97-932) and by the German Research Foundation (DFG, Deutsche Forschungsgemeinschaft) as part of Germany's Excellence Strategy—EXC 2050/1—Project ID 390696704—Cluster of Excellence Center for Tactile Internet with Human-in-the-Loop (CeTI) of Technische Universität Dresden. Open Access Funding by the Publication Fund of the TU Dresden.

## Conflict of Interest

The author declares that the research was conducted in the absence of any commercial or financial relationships that could be construed as a potential conflict of interest.

## Publisher's Note

All claims expressed in this article are solely those of the authors and do not necessarily represent those of their affiliated organizations, or those of the publisher, the editors and the reviewers. Any product that may be evaluated in this article, or claim that may be made by its manufacturer, is not guaranteed or endorsed by the publisher.
